# SorGSD: a sorghum genome SNP database

**DOI:** 10.1186/s13068-015-0415-8

**Published:** 2016-01-07

**Authors:** Hong Luo, Wenming Zhao, Yanqing Wang, Yan Xia, Xiaoyuan Wu, Limin Zhang, Bixia Tang, Junwei Zhu, Lu Fang, Zhenglin Du, Wubishet A. Bekele, Shuaishuai Tai, David R. Jordan, Ian D. Godwin, Rod J. Snowdon, Emma S. Mace, Hai-Chun Jing, Jingchu Luo

**Affiliations:** Genomics and Molecular Breeding of Biofuel Crops, Key Laboratory of Plant Resources, Institute of Botany, Chinese Academy of Sciences, 100093 Beijing, China; Beijing Institute of Genomics, Chinese Academy of Sciences, 100101 Beijing, China; Department of Plant Breeding, Justus Liebig University, Giessen, Germany; BGI-Shenzhen, 518083 Shenzhen, China; Queensland Alliance for Agriculture and Food Innovation, The University of Queensland, Warwick, QLD 4370 Australia; School of Agriculture and Food Sciences, The University of Queensland, Brisbane, QLD 4072 Australia; Department of Agriculture, Fisheries & Forestry (DAFF), Warwick, QLD 4370 Australia; College of Life Sciences and State Key Laboratory of Protein and Plant Gene Research, Peking University, 100871 Beijing, China; Laboratory of Bioinformatics, Wageningen University and Research Centre, Wageningen, The Netherlands

**Keywords:** Sorghum, Bio-energy plant, Genome variation, SNPs, Database curation

## Abstract

**Background:**

Sorghum (*Sorghum bicolor*) is one of the most important cereal crops globally and a potential energy plant for biofuel production. In order to explore genetic gain for a range of important quantitative traits, such as drought and heat tolerance, grain yield, stem sugar accumulation, and biomass production, via the use of molecular breeding and genomic selection strategies, knowledge of the available genetic variation and the underlying sequence polymorphisms, is required.

**Results:**

Based on the assembled and annotated genome sequences of *Sorghum bicolor* (v2.1) and the recently published sorghum re-sequencing data, ~62.9 M SNPs were identified among 48 sorghum accessions and included in a newly developed sorghum genome SNP database SorGSD (http://sorgsd.big.ac.cn). The diverse panel of 48 sorghum lines can be classified into four groups, improved varieties, landraces, wild and weedy sorghums, and a wild relative *Sorghum propinquum*. SorGSD has a web-based query interface to search or browse SNPs from individual accessions, or to compare SNPs among several lines. The query results can be visualized as text format in tables, or rendered as graphics in a genome browser. Users may find useful annotation from query results including type of SNPs such as synonymous or non-synonymous SNPs, start, stop of splice variants, chromosome locations, and links to the annotation on Phytozome (www.phytozome.net) sorghum genome database. In addition, general information related to sorghum research such as online sorghum resources and literature references can also be found on the website. All the SNP data and annotations can be freely download from the website.

**Conclusions:**

SorGSD is a comprehensive web-portal providing a database of large-scale genome variation across all racial types of cultivated sorghum and wild relatives. It can serve as a bioinformatics platform for a range of genomics and molecular breeding activities for sorghum and for other C_4_ grasses.

## Background

Sorghum (*Sorghum bicolor*) originated from Africa and became an important cereal crop after a long period of domestication and selective breeding [1]. Nowadays, it feeds over 500 million people in 98 countries [2], with an estimation of 42 million hectares of cultivated area and 62 million tons of yield per year (FAOSTAT data 2013, http://faostat3.fao.org). In contrast to C_3_ crops such as rice and wheat, sorghum has the C_4_ photosynthetic pathway, which leads to higher photosynthetic efficiency under circumstances of intense light, high temperature and low water supply [2–4]. As such, sorghum has remarkable drought and heat tolerance, and can produce high yield and biomass in areas of harsh conditions with low inputs. Sorghum is not only used for food, but also cultivated with other important economic impacts for forage, sugars and biomass. Furthermore, in recent years sorghum has been regarded as a promising bioenergy feedstock [5], which is comparable to other important biofuel grasses such as maize, sugarcane, *Miscanthus* and switch grass [6, 7]. Moreover, the compact genome and high degree of genetic synteny to other C_4_ grasses make sorghum a potential genetic model for the design of bioenergy crops [8, 9].

Sorghum’s genome is relatively small (~730 M) and simple (10 chromosomes, diploid) compared to other C_4_ crops in the *Poaceae* subfamily, such as maize and sugarcane. The recent completion and availability of a whole genome reference sequence, based on the elite line BTx623, has accelerated the pace of genetic and genomic research in sorghum [10]. The genetic basis of a range of important agronomic traits in sorghum has been elucidated, including drought tolerance and maturity [2]. Nevertheless, to better understand the genetic basis for the considerable phenotypic variation observed in many more agronomic and bioenergy traits of different sorghum accessions, it is necessary to have insight into genomic variation including single nucleotide polymorphisms (SNPs), insertions/deletions (INDELs) and structure variation (SV).

Recently, various high throughput strategies have been developed for genome re-sequencing [11–13], resulting in a large amount of SNP data being generated for sorghum [14–18]. These SNP data, representing high density biomarkers, are a valuable resource for researchers to perform genetic and breeding studies, such as genotyping by sequencing (GBS) [19–21], bulked segregant analysis (BSA) [22], and genome-wide association studies (GWAS) [18, 23, 24]. These studies will not only lead to the highly efficient discovery of key QTLs or genes relevant to important traits, but also contribute to the understanding of the evolutionary relationship of cultivated and wild *Sorghum* species and subspecies.

To enhance the utility of sorghum SNP data, we developed a web-based large-scale genome variation database (SorGSD, http://sorgsd.big.ac.cn). SorGSD contains ~62.9 million SNPs from a diverse panel of 48 sorghum accessions divided into four groups, including improved inbreds, landraces, wild/weedy sorghums, and accessions of the wild relative *Sorghum propinquum*. These SNP data have been annotated and an easy-to-use web interface has been designed for users to browse, search and analyze the SNPs efficiently. SorGSD allows users to query the SNP information and their relevant annotations for individual samples. The search results can be visualized graphically in a genome browser or displayed in formatted tables. Users can also compare SNP data between two and more sorghum accessions. The output of query results can be downloaded for further investigation, or users can bulk download the entire SNP dataset of 48 accessions. SorGSD also manages additional sorghum related information, such as general descriptions of sorghum and its genome, sorghum research institutions around the world, and lists of sorghum literature references.

## Result and discussion

### Database content

**Fig. 1:**
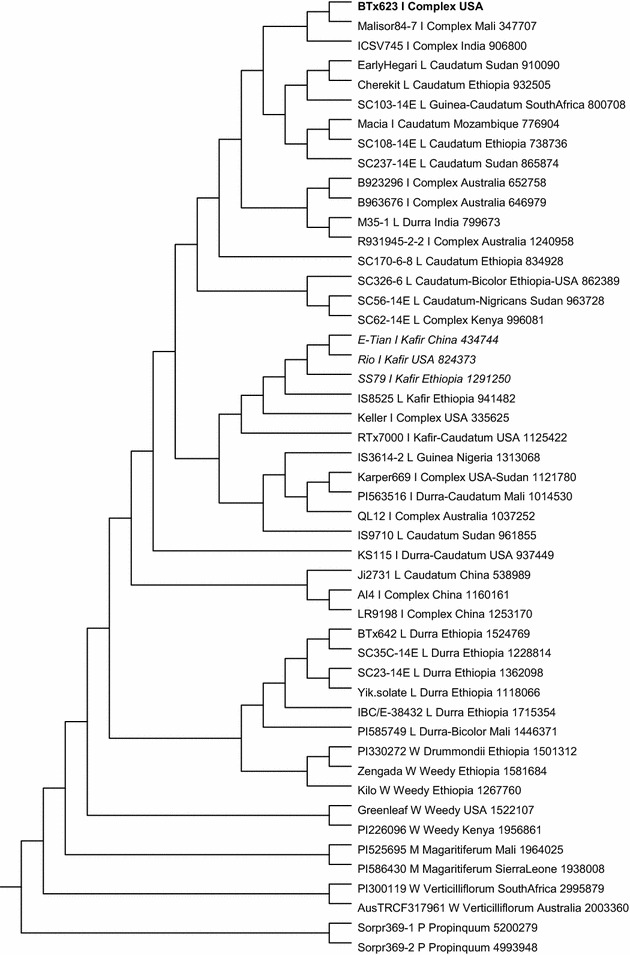
A dendrogram showing the phylogenetic relationships among the diverse set of sorghum lines. Each sample is labelled as follows; the genotype name, sample type (coded, as detailed below), racial type, geographic origin, and total number of SNPs identified. Sample type codes: *I* improved variety, *L* landrace, *W* weedy or wild, *M* margaritiferum, *P* *Sorghum propinquum*. The sorghum reference genome BTx623 is shown in *bold*, sweet sorghums are in *italic*. (Adapted from Mace et al. [16] and redrawn using the tool “Display Newick Trees” under MEGA 6.0, SS79 was added based on the output results of the SNPhylo program [34] using the SNP data.)

SorGSD contains ~62.9 million SNPs identified from the re-sequencing data of 48 sorghum lines mapped to the reference genome BTx623. These sorghum lines represent major cultivated races grouped into landraces or improved varieties, and weedy or wild subspecies. Figure 1 shows the phylogenetic relationship among these sorghum lines [16], with the genotype name and group indicated. Racial type and geographic origin are also included. Additionally, the total number of SNPs identified per sample is indicated. The two *margaritiferum* cultivars (PI525695 M Margaritiferum Mali 1964025 and PI586430 M Margaritiferum Sierra Leone 1938008) are separated into a distinct group since they are highly divergent from other *S. bicolor* races (Fig. 1). Two samples of the allopatric Asian species *Sorghum propinquum* are clustered within a distant group as the outgroup.

The SNP numbers of each sample give an overview of the genomic difference between the reference genome BTx623 and individual genomes. Detailed information about distribution of SNPs in different genomic regions, including genic, intergenic, and intronic regions is provided (Table 1). For genic regions, SNPs found in specific positions such as start and stop codons, splice donator and acceptor sites are listed (Table 2).

**Table 1:** Distribution of SNPs in different genomic regions in 48 sorghum accessions

Genotype	Type	Racial type	Geographic origin	Total SNP numbers						
				All	Intergenic	5′ UTR	Intronic	Non-syn	Syn	3′ UTR
BTx623	I	Complex	USA	0	0	0	0	0	0	0
Malisor84-7	I	Complex	Mali	347707	284944	2079	36036	7834	7175	8261
ICSV745	I	Complex	India	906800	762772	6166	81888	17476	15943	19300
EarlyHegari	L	Caudatum	Sudan	910090	748915	6893	90114	20375	19267	20719
Cherekit	L	Caudatum	Ethiopia	932505	763491	7179	96419	19765	18989	22799
SC103-14E	L	Guinea-Caudatum	South Africa	800708	657087	5589	82356	17216	15899	19275
Macia	I	Caudatum	Mozambique	776904	632772	5698	84057	16103	15199	19937
SC108-14E	L	Caudatum	Ethiopia	738736	600093	5969	78205	16647	15926	18758
SC237-14E	L	Caudatum	Sudan	865874	708878	7299	87805	18947	18174	21154
B923296	I	Complex	Australia	652758	537078	4312	66567	13219	12591	16395
B963676	I	Complex	Australia	646947	521677	5095	71404	14277	14007	17587
M35-1	L	Durra	India	799673	659631	5629	81167	15727	15239	19067
R931945-2-2	I	Complex	Australia	1240958	1045243	10365	109678	22904	21989	26306
SC170-6-8	L	Caudatum	Ethiopia	834928	698227	6107	77492	16224	15499	18180
SC326-6	L	Caudatum-Bicolor	Ethiopia-USA	862389	702263	6869	91952	18410	17529	21739
SC56-14E	L	Caudatum-Nigricans	Sudan	963728	788668	7831	98457	21451	19783	23541
SC62-14E	L	Complex	Kenya	996081	803234	8304	108872	23186	21516	26629
E-Tian	I	Kafir	China	434744	323422	8666	45605	19683	20023	14334
Rio	I	Kafir	USA	824373	660153	7410	92526	19751	18890	21916
SS79	I	Kafir	Ethiopia	1291250	1048752	16348	122623	34079	32586	30973
IS8525	L	Kafir	Ethiopia	941482	777365	7926	92487	19527	18210	22188
Keller	I	Complex	USA	335625	238622	4096	50617	13143	12560	14148
RTx7000	I	Kafir-Caudatum	USA	1125422	943142	9873	102492	21075	19846	24795
IS3614-2	L	Guinea	Nigeria	1313068	1102724	8066	123657	22931	21749	29188
Karper669	I	Complex	USA-Sudan	1121780	935393	7839	106347	22061	20738	25193
PI563516	I	Durra-Caudatum	Mali	1014530	835382	8632	100645	20999	20101	24679
QL12	I	Complex	Australia	1037252	860948	7376	101401	20297	18933	24245
IS9710	L	Caudatum	Sudan	961866	783299	6937	102930	20584	20071	24013
KS115	I	Durra-Caudatum	USA	937449	767552	4773	102892	17830	16454	24350
Ji2731	L	Caudatum	China	538989	395020	10246	60269	24847	25652	19250
AI4	I	Complex	China	1160161	963494	7757	112978	22722	22193	26553
LR9198	I	Complex	China	1253170	1039361	9778	121486	24483	23565	29609
BTx642	L	Durra	Ethiopia	1524769	1287876	12862	132322	27541	26021	32749
SC35C-14E	L	Durra	Ethiopia	1228814	1028072	7766	115689	23108	22143	27565
SC23-14E	L	Durra	Ethiopia	1362098	1146377	9130	123135	24986	23680	29949
Yik.solate	L	Durra	Ethiopia	1118066	933012	5181	116030	17059	15380	27540
IBC/E-38432	L	Durra	Ethiopia	1715354	1430193	11247	167795	30353	29061	40485
PI585749	L	Durra-Bicolor	Mali	1446371	1210097	11321	133531	27449	25917	32590
PI330272	W	Drummondii	Ethiopia	1501312	1242394	10899	147465	30448	29048	35194
Zengada	W	Weedy	Ethiopia	1581684	1315478	10824	155882	28624	27247	37720
Kilo	W	Weedy	Ethiopia	1267760	1047467	5627	137344	21473	19909	31449
Greenleaf	W	Weedy	USA	1522107	1268287	10468	145993	29247	28130	34204
PI226096	W	Weedy	Kenya	1956801	1641444	16255	179268	35250	33730	43508
PI525695	M	Margaritiferum	Mali	1964025	1628455	12730	197292	36202	35569	46286
PI586430	M	Margaritiferum	Sierra Leone	1938008	1594348	13766	198477	38894	38431	46271
PI300119	W	Verticilliflorum	South Africa	2995879	2482294	26648	290919	56213	56617	71315
AusTRCF317961	W	Verticilliflorum	Australia	2003360	1625419	12596	226288	38953	39283	52566
Sorpr369-1	P	Propinquum	–	5200279	3971685	58105	713492	124517	141591	163430
Sorpr369-2	P	Propinquum	–	4993948	3794524	53315	704812	118631	135432	160696

*I* improved variety, *L* landrace, *W* wild/weedy, *M* *margaritiferum*, *P* *Sorghum propinquum*

**Table 2:** **Distribution of major effect SNPs in different genic sites and regions in 48 sorghum accessions**

Genotype	Type	Racial type	Geographic origin	Start codon			Stop codon			Splice sites		
				Gain	Lost	Variant	Gain	Lost	Retain	Donor	Acceptor	Region
BTx623	I	Complex	USA	0	0	0	0	0	0	0	0	0
Malisor84-7	I	Complex	Mali	380	16	1	130	39	10	28	32	742
ICSV745	I	Complex	India	1084	45	9	246	64	21	69	74	1643
EarlyHegari	L	Caudatum	Sudan	1281	35	12	282	81	18	69	69	1960
Cherekit	L	Caudatum	Ethiopia	1283	42	12	291	68	18	66	72	2011
SC103-14E	L	Guinea-Caudatum	South Africa	1014	39	8	244	72	20	51	75	1763
Macia	I	Caudatum	Mozambique	996	38	5	239	62	26	51	60	1661
SC108-14E	L	Caudatum	Ethiopia	1054	42	9	230	60	20	51	68	1604
SC237-14E	L	Caudatum	Sudan	1242	50	6	282	79	22	57	72	1807
B923296	I	Complex	Australia	770	26	9	199	53	19	53	72	1395
B963676	I	Complex	Australia	962	30	5	192	62	18	57	64	1510
M35-1	L	Durra	India	1048	39	5	246	68	21	66	74	1646
R931945-2-2	I	Complex	Australia	1703	74	15	331	81	29	88	110	2042
SC170-6-8	L	Caudatum	Ethiopia	1065	44	5	245	69	27	55	58	1631
SC326-6	L	Caudatum-Bicolor	Ethiopia-USA	1220	34	10	266	82	26	62	96	1831
SC56-14E	L	Caudatum-Nigricans	Sudan	1433	40	10	328	78	24	58	80	1946
SC62-14E	L	Complex	Kenya	1455	38	10	295	99	28	71	94	2250
E-Tian	I	Kafir	China	1430	41	14	228	65	25	57	66	1085
Rio	I	Kafir	USA	1273	47	16	259	72	21	65	75	1899
SS79	I	Kafir	Ethiopia	2602	78	21	419	116	37	102	130	2384
IS8525	L	Kafir	Ethiopia	1353	46	17	261	69	24	64	87	1858
Keller	I	Complex	USA	750	27	7	212	48	12	36	45	1302
RTx7000	I	Kafir-Caudatum	USA	1605	47	15	284	93	32	78	97	1948
IS3614-2	L	Guinea	Nigeria	1421	48	13	358	95	37	83	112	2586
Karper669	I	Complex	USA-Sudan	1362	52	11	301	93	24	63	94	2209
PI563516	I	Durra-Caudatum	Mali	1427	53	13	298	86	27	62	94	2032
QL12	I	Complex	Australia	1321	45	10	313	79	25	64	98	2097
IS9710	L	Caudatum	Sudan	1265	38	10	301	85	21	73	78	2161
KS115	I	Durra-Caudatum	USA	900	35	12	270	77	33	71	89	2111
Ji2731	L	Caudatum	China	1666	52	13	265	76	23	74	62	1474
AI4	I	Complex	China	1416	45	12	291	78	24	90	99	2409
LR9198	I	Complex	China	1735	47	10	331	95	27	103	107	2433
BTx642	L	Durra	Ethiopia	2114	75	23	363	107	38	93	99	2486
SC35C-14E	L	Durra	Ethiopia	1402	48	16	317	89	32	84	94	2389
SC23-14E	L	Durra	Ethiopia	1587	55	14	384	107	31	87	108	2468
Yik.solate	L	Durra	Ethiopia	990	25	8	249	67	28	79	90	2328
IBC/E-38432	L	Durra	Ethiopia	1965	70	14	442	113	45	108	121	3342
PI585749	L	Durra-Bicolor	Mali	1930	65	17	388	109	43	95	130	2689
PI330272	W	Drummondii	Ethiopia	1865	56	11	458	123	49	100	148	3054
Zengada	W	Weedy	Ethiopia	1864	59	13	413	111	45	95	147	3162
Kilo	W	Weedy	Ethiopia	1058	35	5	294	73	32	85	106	2803
Greenleaf	W	Weedy	USA	1871	60	16	411	122	34	110	116	3038
PI226096	W	Weedy	Kenya	2767	76	16	495	145	48	148	148	3503
PI525695	M	Margaritiferum	Mali	2318	73	15	524	135	46	136	162	4082
PI586430	M	Margaritiferum	Sierra Leone	2525	82	15	562	144	47	138	175	4133
PI300119	W	Verticilliflorum	South Africa	4441	132	29	786	204	90	211	224	5756
AusTRCF317961	W	Verticilliflorum	Australia	2278	80	16	521	145	53	163	185	4814
Sorpr369-1	P	Propinquum	–	9859	249	42	1519	378	236	407	481	14288
Sorpr369-2	P	Propinquum	–	9169	241	41	1437	359	240	405	465	14181

*I* improved variety; *L* landrace; *W* wild/weedy; *M* *margaritiferum*; *P Sorghum propinquum*

All the SNP data shown in the two tables can be easily accessed either as statistical information through the Help page of the database, or through the user interface. The original data of sequencing short reads, the assembled sequence and the SNP data of each accession can be downloaded.

### User interface

SorGSD offers three main functions (search, compare and browse), for users to search, display and retrieve the SNPs and their annotations.

**Fig. 2:**
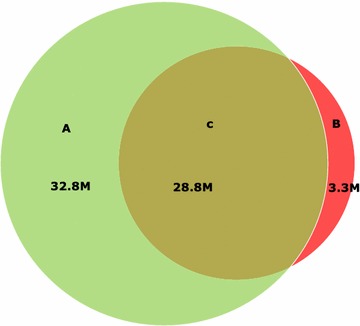
Venn diagram of SNPs identified by two pipelines. A SNPs called by the GATK-based pipeline. B SNPs called by the SOAPsnp- and realSFS-based pipeline. C The set of highly reliable SNPs those were identified by both pipelines

The search function provides a user-friendly web interface to query SNP information. Users can search SNPs by specifying chromosomal co-ordinates or the locus ID. Users can also query SNPs based on their genotypes, and predicted variant effects. In addition, users can compare the SNPs between two and more sorghum lines. The query results can be shown as a formatted table which contains the information of ID, chromosome position, genomic location and predicted coding effects, 5′ and 3′ flanking sequences, reference and derived alleles, respectively. SNPs from the stringent set identified by both pipelines (see description in “Methods” and Fig. 2 for details) are highlighted with a green background in the result page. The output of the query results can be downloaded as flat text or formatted tables for further investigation.

SorGSD also provides several data browsing functionalities under the “Browse” pull-down menu. The “Total SNPs” tab lists the SNP numbers on 10 chromosomes of all 48 accessions. Users can select a group, e.g. Landraces, to display the SNP numbers of these accessions within this group. Mouse-clicking these SNP numbers will bring up the list of SNPs of a specific accession. Given that the different location in genes such as coding regions, as well as the non-synonymous information are often of great interest for further study, the “Genic SNP” tab lists several submenus including “Coding SNP”, “Synonymous SNP”, and “Non-synonymous SNP” so that information can be tailored to user requirements.

The “Browse on Chromosome” tab leads to an interactive graphic window to visualize SNPs in a genome browser. Users can customize the visualization interface by selecting different data types, including SNPs, genes, transcripts, allele frequencies, and the SNP density information. Users can obtain a pie chart showing the allele frequency, SNP density in 300 kb windows size, related gene and transcript information.

### Help information

SorGSD provides a help resource for users to better access the SNP data, as well as proving links to additional sorghum research related resources.

The help menu provides a “How to” page, which gives a number of examples for users to learn how to search and compare target SNPs. For example, a step-by-step user-guide shows how to obtain non-synonymous SNPs in chromosome 1 of sweet sorghum E-Tian, and how to compare SNPs between sweet sorghum E-Tian and two grain sorghum Ji2731 and Keller. An FAQs page provides answers to a range of frequently asked questions not only about the content and usage of SorGSD but more broadly about sorghum genomics. Detailed information including software tools, parameters and data sources is presented in the “Pipeline” page. The “Statistics” page shows the SNP numbers distributed in different genomic regions (Table 1) and specific genic sites (Table 2). The “Data source” page shows the general information of 48 sorghum lines, including their geographic origins, and links to the US Germplasm Resources Information Network (http://www.ars-grin.gov).

The “About” tab contains several pages related to sorghum research. The Sorghum Genome page provides a brief introduction to the reference genome BTx623, including genome size and gene number. The Resource page provides links to online databases, research institutions, sorghum producers and handbooks. The reference page lists selected recently published papers in the fields of sorghum genomics, genetics, QTLs, etc., with links to full lists in PubMed.

## Conclusions and future directions

High coverage resequencing data from two previous sorghum studies [15, 16] were used to identify SNPs among 48 sorghum genotypes by combining three SNP calling tools and updating the SNPs datasets using the sorghum reference annotation (Version 2.1). In addition, we annotated the effect of SNP variants on genes of each sorghum accession. SorGSD has already received over two thousands of visits from more than 30 countries around the world since it went online a few months ago. During the review process of this manuscript, we were happy to know that a new website Sorghum Genomics (https://www.purdue.edu/sorghumgenomics) developed at Purdue University became available as a functional gene discovery platform.

We will improve the SNP calling pipeline and the annotation procedure to obtain more accurate SNP data and upload them into the database. Furthermore, we will include additional types of genome variation data detected by newly developed pipelines, including INDELs and copy number variations (CNVs). At the same time, we will improve the web interface especially in the search function and give more examples in the user guide to help novice users to access the database easily. We will add more analytical functionalities so that users can perform more analyses such as Blast search, sequence alignment and phylogenetic analysis.

SorGSD can serve as a bioinformatics platform to inform wet-lab experiments including biomarker development, allele mining and gene function assessment. In addition to the collaboration among research groups involving in this work, we will collaborate with other domestic and international laboratories in the sorghum research community to sequence and annotate more sorghum accessions in the future.

We will update the database regularly and add SNP datasets with newly available re-sequenced sorghum accessions. We hope that the high density of these SNP data at genomic level collected from the major races of cultivated sorghum as well as other subspecies is a rich repository for a broader research community working in biomarker identification, genetic analysis and molecular breeding, especially for energy plant sweet sorghum cultivation.

## Methods

The construction of SorGSD was a multi-step process. Firstly, the sorghum re-sequencing paired-end raw reads reported in the previously published works were downloaded [15, 16]. In addition, the paired-end raw reads generated in-house for a sweet sorghum line SS79 were included [unpublished data]. Secondly, the raw reads were mapped to the reference sorghum genome (BTx623) [10] using the BWA program [25]. SNPs were identified using the software GATK [26, 27], realSFS (http://popgen.dk/angsd/index.php/RealSFS) and SOAPsnp [28] and annotated using SnpEff [29]. With the SNP matrix finalized, a web interface was designed for users to browse and search the SNPs and related annotations. Details for the database construction are described as follows and are also available on the designated website.

### Data source

The raw reads of sequencing data were from three original datasets. The largest dataset [16] contains 44 sorghum accessions and represent the major races of cultivated sorghum as well as their wild relatives. The second dataset [15] contains three accessions of cultivated sorghums. The raw reads of these two datasets can be downloaded from the NCBI sequence read archive (SRA) (accessions SRS378430-SRS378473, and accessions SRX100115-SRX100138). The third dataset contains the paired-end reads of sorghum line SS79, a cultivated sweet sorghum inbred. These data were recently generated in our laboratory using an Illumina HiSeq 2000 platform with insert size of 500 bp and have not been submitted to NCBI. The average sequencing depth of all sorghum accessions is about 20×, ranging from 12 to 54×.

### SNP calling pipeline

After trimming adapters, the clean reads were mapped to version 2.1 of the reference genome (available via http://phytozome.jgi.doe.gov/pz/portal.html#!info?alias=Org_Sbicolor) using the BWA program [25], allowing a maximum of five mismatches and disabling long gaps in the mapping procedure. The average counts of the mapping rate, the unique mapping rate and the mapping coverage were 0.957, 0.681 and 0.881 respectively, excluding the two *S. propinquum* accessions. The SAM tools package [30] was used to convert mapping results to BAM format, and then the Picard program (http://picard.sourceforge.net) was applied to eliminate duplicated reads generated during the process of library construction.

Subsequently, the GATK tools [26, 27] were used to recalibrate the base quality score to obtain more accurate quality scores for each base and realign reads around known INDELs. The refined data from all individuals were jointly used to call a raw SNPs set by GATK HaplotypeCaller. Finally, a set of SNPs were identified, using the variant quality score to recalibrate the procedure in GATK. In total, we identified 62,888,582 SNPs across all 48 sorghum lines, corresponding to 15,357,261 sites in the reference genome. The GATK based SNP calling pipeline is similar to that reported in a recent publication [31]. SNPs were additionally identified using the pipeline described previously using realSFS (http://popgen.dk/angsd/index.php/RealSFS) and SOAPsnp [28], described by Mace et al. [16]. Approximately 28 million highly stringent SNPs were in common between the two SNP identification pipelines (Fig. 2) with the GATK-based pipeline identifying more SNPs than the SOAPsnp-based pipeline. The total number of SNPs called by the GATK based pipeline was found to be comparable to the study by Evans et al. [32], which employed the CLC Workbench software (CLC Bio-Qiagen, Aarhus, Denmark). All the SNPs identified by the GATK pipeline were stored in SorGSD, with the subset of 28 million highly stringent SNPs highlighted in the results page. Finally, the effect of variants on all the v2.1 predicted gene models for each sorghum accession were predicted and annotated using the SnpEff program (version 4.0e) [29].

### Database implementation

The SNP data and their related annotations were formatted into tables and stored in SorGSD using the MySQL database management system (version 5). The web interface of SorGSD was designed by JAVA/JSP (JDK 1.6) under the Apache/Tomcat web server (version 2.0) running under a Linux operation system (CentOS 6). We installed the generic genome browser GBrowse [33] as a chromosome-based visualization tool to display these genomic SNPs and annotations.

## Abbreviations

SNP: single nucleotide polymorphism
INDEL: insertion/deletion
SV: structure variation
GBS: genotyping by sequencing
BSA: bulked segregant analysis
GWAS: genome-wide association study
QTLs: quantitative trait locus
CNV: copy number variation

## References

[1] Doggett H (1967). Yield increase from sorghum hybrids. Nature.

[2] Pennisi E (2009). Plant genetics: how sorghum withstands heat and drought. Science.

[3] Osborne CP, Beerling DJ (2006). Nature’s green revolution: the remarkable evolutionary rise of C4 plants. Philos Trans R Soc Lond B Biol Sci.

[4] Sasaki T, Antonio BA (2009). Plant genomics: sorghum in sequence. Nature.

[5] Rooney WL, Blumenthal J, Bean B, Mullet JE (2007). Designing sorghum as a dedicated bioenergy feedstock. Biofuels, Bioprod Biorefin.

[6] Carpita NC, McCann MC (2008). Maize and sorghum: genetic resources for bioenergy grasses. Trends Plant Sci.

[7] Vermerris W (2011). Survey of genomics approaches to improve bioenergy traits in maize, sorghum and sugarcane free access. J Integr Plant Biol.

[8] Calviño M, Messing J (2012). Sweet sorghum as a model system for bioenergy crops. Curr Opin Biotechnol.

[9] Mullet J, Morishige D, McCormick R, Truong S, Hilley J, McKinley B, Anderson R, Olson SN, Rooney W (2014). Energy sorghum—a genetic model for the design of C4 grass bioenergy crops. J Exp Bot.

[10] Paterson AH, Bowers JE, Bruggmann R, Dubchak I, Grimwood J, Gundlach H, Haberer G, Hellsten U, Mitros T, Poliakov A (2009). The Sorghum bicolor genome and the diversification of grasses. Nature.

[11] Elshire RJ, Glaubitz JC, Sun Q, Poland JA, Kawamoto K, Buckler ES, Mitchell SE (2011). A Robust, Simple genotyping-by-sequencing (GBS) approach for high diversity species. PLoS One.

[12] Davey JW, Hohenlohe PA, Etter PD, Boone JQ, Catchen JM, Blaxter ML (2011). Genome-wide genetic marker discovery and genotyping using next-generation sequencing. Nat Rev Genet.

[13] Wang S, Meyer E, McKay JK, Matz MV (2012). 2b-RAD: a simple and flexible method for genome-wide genotyping. Nat Meth.

[14] Nelson JC, Wang S, Wu Y, Li X, Antony G, White FF, Yu J (2011). Single-nucleotide polymorphism discovery by high-throughput sequencing in sorghum. BMC Genom.

[15] Zheng L-Y, Guo X-S, He B, Sun L-J, Peng Y, Dong S-S, Liu T-F, Jiang S, Ramachandran S, Liu C-M, Jing H-C (2011). Genome-wide patterns of genetic variation in sweet and grain sorghum (Sorghum bicolor). Genome Biol.

[16] Mace ES, Tai S, Gilding EK, Li Y, Prentis PJ, Bian L, Campbell BC, Hu W, Innes DJ, Han X (2013). Whole-genome sequencing reveals untapped genetic potential in Africa’s indigenous cereal crop sorghum. Nature Commun..

[17] Bekele WA, Wieckhorst S, Friedt W, Snowdon RJ (2013). High-throughput genomics in sorghum: from whole-genome resequencing to a SNP screening array. Plant Biotechnol J.

[18] Morris GP, Ramu P, Deshpande SP, Hash CT, Shah T, Upadhyaya HD, Riera-Lizarazu O, Brown PJ, Acharya CB, Mitchell SE (2012). Population genomic and genome-wide association studies of agroclimatic traits in sorghum. Proc Natl Acad Sci.

[19] Nielsen R, Paul JS, Albrechtsen A, Song YS (2011). Genotype and SNP calling from next-generation sequencing data. Nat Rev Genet.

[20] Spindel J, Wright M, Chen C, Cobb J, Gage J, Harrington S, Lorieux M, Ahmadi N, McCouch S (2013). Bridging the genotyping gap: using genotyping by sequencing (GBS) to add high-density SNP markers and new value to traditional bi-parental mapping and breeding populations. Theor Appl Genet.

[21] Morishige D, Klein P, Hilley J, Sahraeian SM, Sharma A, Mullet J (2013). Digital genotyping of sorghum— a diverse plant species with a large repeat-rich genome. BMC Genom.

[22] Han Y, Lv P, Hou S, Li S, Ji G, Ma X, Du R, Liu G (2015). Combining next generation sequencing with bulked segregant analysis to fine map a stem moisture locus in sorghum (*Sorghum bicolor* L. Moench). PLoS ONE.

[23] Rhodes DH, Hoffmann L, Rooney WL, Ramu P, Morris GP, Kresovich S (2014). Genome-wide association study of grain polyphenol concentrations in global sorghum [*Sorghum bicolor* (L.) Moench] germplasm. J Agric Food Chem.

[24] Adeyanju A, Little C, Yu J, Tesso T (2015). Genome-wide association study on resistance to stalk rot diseases in grain sorghum. G3 (Bethesda).

[25] Li H, Durbin R (2010). Fast and accurate long-read alignment with Burrows–Wheeler transform. Bioinformatics.

[26] McKenna A, Hanna M, Banks E, Sivachenko A, Cibulskis K, Kernytsky A, Garimella K, Altshuler D, Gabriel S, Daly M, DePristo MA (2010). The genome analysis toolkit: a Mapreduce framework for analyzing next-generation DNA sequencing data. Genome Res.

[27] DePristo MA, Banks E, Poplin R, Garimella KV, Maguire JR, Hartl C, Philippakis AA, del Angel G, Rivas MA, Hanna M (2011). A framework for variation discovery and genotyping using next-generation DNA sequencing data. Nat Genet.

[28] Li R, Li Y, Fang X, Yang H, Wang J, Kristiansen K (2009). SNP detection for massively parallel whole-genome resequencing. Genome Res.

[29] Cingolani P, Platts A, Wang LL, Coon M, Nguyen T, Wang L, Land SJ, Lu X, Ruden DM (2012). A program for annotating and predicting the effects of single nucleotide polymorphisms, SnpEff: SNPs in the genome of *Drosophila melanogaster* strain w1118; iso-2; iso-3. Fly (Austin)..

[30] Li H, Handsaker B, Wysoker A, Fennell T, Ruan J, Homer N, Marth G, Abecasis G, Durbin R, Subgroup GPDP (2009). The sequence alignment/map format and SAM tools. Bioinformatics.

[31] McCormick RF, Truong SK, Mullet JE (2015). RIG: recalibration and interrelation of genomic sequence data with the GATK. G3 (Bethesda).

[32] Evans J, McCormick RF, Morishige D, Olson SN, Weers B, Hilley J, Klein P, Rooney W, Mullet J (2013). Extensive variation in the density and distribution of DNA polymorphism in sorghum genomes. PLoS One.

[33] Stein LD, Mungall C, Shu S, Caudy M, Mangone M, Day A, Nickerson E, Stajich JE, Harris TW, Arva A, Lewis S (2002). The generic genome browser: a building block for a model organism system database. Genome Res.

[34] Lee T-H, Guo H, Wang X, Kim C, Paterson AH (2014). SNPhylo: a pipeline to construct a phylogenetic tree from huge SNP data. BMC Genom.

