# Erratum to: SorGSD: a sorghum genome SNP database

**DOI:** 10.1186/s13068-016-0450-0

**Published:** 2016-02-15

**Authors:** Hong Luo, Wenming Zhao, Yanqing Wang, Yan Xia, Xiaoyuan Wu, Limin Zhang, Bixia Tang, Junwei Zhu, Lu Fang, Zhenglin Du, Wubishet A. Bekele, Shuaishuai Tai, David R. Jordan, Ian D. Godwin, Rod J. Snowdon, Emma S. Mace, Jingchu Luo, Hai-Chun Jing

**Affiliations:** Genomics and Molecular Breeding of Biofuel Crops, Key Laboratory of Plant Resources, Institute of Botany, Chinese Academy of Sciences, 100093 Beijing, China; Beijing Institute of Genomics, Chinese Academy of Sciences, 100101 Beijing, China; Department of Plant Breeding, Justus Liebig University, Giessen, Germany; BGI-Shenzhen, 518083 Shenzhen, China; Queensland Alliance for Agriculture and Food Innovation, The University of Queensland, Warwick, QLD 4370 Australia; School of Agriculture and Food Sciences, The University of Queensland, Brisbane, QLD 4072 Australia; Department of Agriculture and Fisheries (DAF), The University of Queensland, Warwick, QLD 4370 Australia; College of Life Sciences and State Key Laboratory of Protein and Plant Gene Research, Peking University, 100871 Beijing, China; Laboratory of Bioinformatics, Wageningen University and Research Centre, Wageningen, The Netherlands

## Erratum to: Biotechnol Biofuels (2016) 9:6 DOI 10.1186/s13068-015-0415-8

After publication of this work [1], we noted that the given order of co-authors was incorrect. The list of authors has now been corrected in this erratum and in the original article. We apologize for any inconvenience this oversight may have caused.
